# Characterization of a VHS virus genotype III isolated from rainbow trout (*Oncorhychus mykiss*) at a marine site on the west coast of Norway

**DOI:** 10.1186/1743-422X-7-19

**Published:** 2010-01-26

**Authors:** Henrik Duesund, Stian Nylund, Kuninori Watanabe, Karl F Ottem, Are Nylund

**Affiliations:** 1 Department of Biology, University of Bergen, Thormohlensgt 55, 5020 Bergen, Norway

## Abstract

**Background:**

Norwegian production of rainbow trout (*Oncorhynchus mykiss*) has been without any outbreaks of VHS for many years until the disease emerged in a farm in western Norway in November 2007. The fish were, in addition to VHS virus, positive for gill chlamydia-like bacteria, *Flavobacterium psychrophilum*, and a microsporidian. A new VHS virus genotype III was isolated from the fish in RTgill-W1 cells and the complete coding region (11,065 nucleotides) was sequenced. This virus was also used in a challenge experiment to see if it could cause any mortality in rainbow trout in sea water.

**Results:**

This is the first time a nearly complete sequence of a genotype III virus isolate has been presented. The organization of the genes is the same as in the other VHS virus genotypes studied (GI and GIV). Between the ORFs are nontranslated regions that contain highly conserved sequences encompassing the polyadenylation signal for one gene, and the putative transcription initiation site of the next gene. The intergenic regions vary in length from 74 nt to 128 nt. The nucleotide sequence is more similar to genotype I isolates compared to isolates from genotype II and IV. Analyses of the sequences of the N and G protein genes show that this new isolate is distinct from other VHS virus isolates and groups closely together with isolates from genotype III. In a challenge experiment, using intraperitoneal (ip) injection of the isolate, co-habitation with infected fish, and bath challenge, mortalities slightly above 40% were obtained. There was no significant difference in mortality between the bath challenged group and the ip injected group, while the mortality in the co-habitation group was as low as 30%.

**Conclusions:**

All VHS virus isolates in genotype III are from marine fish in the North East Atlantic. Unlike the other known genotype III isolates, which are of low virulence, this new isolate is moderately virulent. It was not possible to detect any changes in the virus genome that could explain the higher virulence. A major problem for the study of virulence factors is the lack of information about other genotype III isolates.

## Background

Viral haemorrhagic septicaemia virus (VHSV) is an enveloped, single stranded, negative-strand RNA virus belonging to the genus *Novirhabdovirus*, family *Rhabdoviridae *[1]. The VHS virus genome consists of approximately 11 k nucleotides and six genes encoding nucleocapsid- (N), phospho- (P), matrix- (M), glyco- (G), non-structural- (Nv) and RNA polymerase (L) protein. Based on phylogenetic analysis of the N, P, G and Nv protein genes the VHS virus isolates have been divided into four different genotypes; VHS virus genotypes I, II, III and IV [2-7]. The third VHS virus genotype (III) represents isolates from marine fish species in Kattegat, Skagerrak and the North Sea [8] and a member of this genotype was in the autumn of 2007 associated with about 10% mortality in a rainbow trout farm in western Norway [9,10]. VHS virus genotype III has been found in eel (*Anguilla anguilla*), cod (*Gadus morhua*), herring (*Clupea harengus*), sprat (*Sprattus sprattus*), haddock (*Melanogrammus aeglefinus*), Norway pout (*Trisopterus esmarkii*), poor cod (*Trisopterus minutus*), blue whiting (*Micromesistius poutassou*), withing (*Merlangius merlangus*), turbot (*Scophthalmus maximus*), greenland halibut (*Reinhardtius hippoglossoides*) and lesser argentine (*Argentina sphyraena*) [cf [3,5,8,11]]. The outbreak of VHS in Norway is the first time an isolate belonging to genotype III is found in rainbow trout.

According to existing literature challenge of rainbow trout with the VHS virus, genotype III, should not result in any significant mortality [12]. However, it is to be expected that viruses which enter into farmed populations of fish may show some virulence and possibly cause mortality. It has been shown that VHS virus belonging to genotype III may cause mortality when challenging turbot [13-15] and halibut [16], which suggests that the susceptibility of the host species is also important for the expected mortality. This has also been observed for other viruses isolated from fish [17,18]. A challenge experiment on rainbow trout fingerlings (10.1 grams) in fresh water, using a VHS virus isolate type III from the same outbreak as the isolate used in this study, has already been carried out resulting in mortality after immersion and injections of 70% and 100%, respectively [9]. However, this genotype III VHS virus is a purely marine virus and experiment on fingerlings in fresh water may not be representative for the susceptibility of larger rainbow trout in sea water, and the resulting mortality in sea water cannot be predicted based on this challenge experiment.

The aim of the present study is to see if this VHS virus genotype III with the first completely characterized coding region may cause any mortality when challenging rainbow trout in full sea water. The genome of this isolate will be compared with partly sequenced VHS virus, genotype III, and completely sequenced coding regions and intergenic regions of other VHS virus, genotypes I and IV. Such a comparison may also give clues as to which changes in the genome may influence the virulence or ability to cause mortality in rainbow trout populations.

## Results

### Genome of isolate FA28.11.07

**Figure 1:**
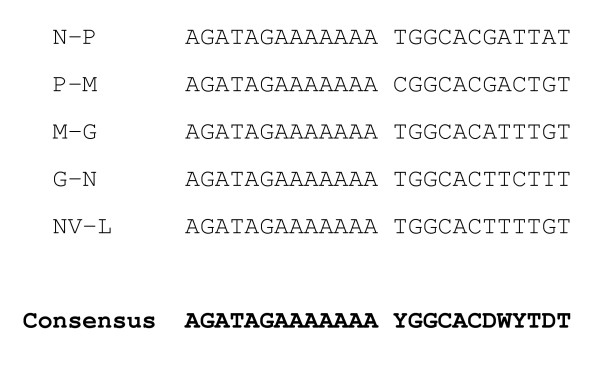
**Comparison of the conserved sequence parts of the intergenic regions within the VHS virus isolate, FA28.11.07, genome**. The sequences between the genes N and P (N-P), P and M (P-M), M and G (M-G), G and NV (G-NV), NV and L (NV-L) are listed in message sense along with the consensus sequence. The sequences consist of a polyadenylation signal and a putative transcription initiation site, respectively.

The first genome of a VHS virus in genotype III, strain FA28.11.07, containing all protein coding sequences (CDS) and intergenic regions (ITRs), has been sequenced (accession no: EU481506). The sequence is 11,065 nucleotides (nt) long and contains six open reading frames (ORF) in the order 3'-N-P-M-G-NV-L-5'. This arrangement is identical to what has been found for other, fully sequenced, VHS virus isolates in genotype I [19,20] and IV (accession no: AB490792). Between the ORFs are nontranslated regions that include highly conserved sequences encompassing the polyadenylation signal for one gene, and the putative transcription initiation site of the next gene. An alignment of the conserved nontranslated sequences is shown in Figure 1. The intergenic regions vary in length, from 74 nt between the G and NV ORFs, to 128 nt between the NV and L ORFs. This is consistent with what has been found for VHS virus isolates in other genotypes except that the length of the intergenic sequences in genotype IV isolates are slightly different. The polyadenylation signal is also present after the ORF of the L protein (21 nt downstream), but the sequence (aga ttg aaa aaa a) is slightly different from that found in the intergenic regions.

**Figure 2:**
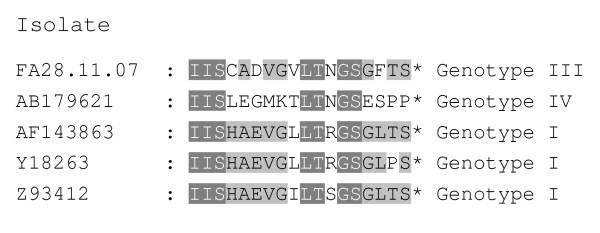
**The CO end (last 18 amino acids) of the deduced NV protein sequence from VHS virus isolates belonging to genotypes I, III and IV**.

Characteristics of the different protein coding genes and their deduced ORFs are listed in table 1. When comparing the amino acid identity of the deduced proteins of isolate FA28.11.07 (genotype III) to that of fully or partially sequenced VHS virus isolates, all proteins share a higher identity to the genotype I isolates than isolates from genotypes II and IV (table 2). The only genes sequenced from other genotype III isolates are those coding for the G and NV proteins and these show the highest identity to FA28.11.07. The short ORF (366 nt), located between the G and L protein, encodes the NV protein which is the most variable protein based on amino acid (aa) sequences. The variation is found throughout the aa sequence, but the latter 14 aa are distinctly different in the three genotypes I, III and IV (Figure 2). Most of the variation in the nucleoprotein (N) of VHS virus isolates, comparing genotypes I, III and IV, is found between aa 37 - 132 and among the last 37 aa in the peptide, avoiding the conserved RNA binding domain suggested to be in the middle region of the protein. The aa variation in the P protein, among VHS virus isolates, is mainly in the first third of the protein, while there is little variation in the M protein.

**Table 1:** VHSV isolate FA28.11.07 genome transcription units and deduced protein products.

	mRNA features (nt)				Deduced protein features (aa) Calculated		
Gene	Length	5'UTR	ORF	3'UTR	Length	Mr	pI
**NP**	1368	113	1212	43	404	44.1	5.2
**P**	761	58	666	37	222	24.5	8.5
**Matrix**	742	83	603	56	201	22.3	9.3
**G**	1610	35	1521	54	507	57.0	6.5
**NV**	423	23	366	34	122	13.6	5.4
**L**	6086	97	5952	37	1984	224.4	7.6

**Table 2:** Pairwise percent amino acid identities of FA28.11.07 proteins with protein sequences in other VHSV isolates.

			% Amino acid identity^a^						
					
Isolate code	Country/origin	Genotype	N	P	M	G	NV	L	Accession no.^b^
DK- Hededam	Denmark	I	92.8	**96.8**	94.5	96.4	84.4	97.7	Z93412
DE- Fil3	Germany	I- a	93.1	96.4	94.0	96.3	81.1	97.7	NC_000855
FR-14-58	France	I- a	**93.6**	95.9	94.0	96.3	84.4	97.6	AF143863
FR-07-71	France	I- a	92.6	94.1	94.5	95.3	82.8	83.8^c^	AJ233396
FR-07-71	France	I- a	-	-	-	-	-	75.9^c^	AJ009814
UK-96-43	England	I-b	93.1	95.5	**95.0**	96.3	84.4	97.6	AF143862
DK-M.rhabdo	Baltic Sea	I- b	**93.6**	95.5	94.5	97.0	90.2	**98.6**	Z93414
DK-2835	Denmark	I- c	-	-	-	95.9	-	-	AY546585
FI-ka66	Gulf of Bothnia	I- d	-	-	-	96.6	-	-	AY546614

DK-1p52	Baltic Sea	II	-	-	-	94.3	70.5	-	AY546576/DQ159194
DK-1p53	Baltic Sea	II	-	-	-	94.1	70.0	-	AY546577/DQ159195

UK-860/94	Scotland	III	-	-	-	97.4	92.6	-	AY546628/DQ159203
UK-H17/2/95	North Sea	III	-	-	-	99.2	**96.*7***	-	AY546629/DQ159202
DK-4p168	Skagerrak	III	-	-	-	**99.8**	-	-	AY546582
UK-MLA98/6PT11	North Sea	III	-	-	-	99.4	-	-	AY546632
DK-4p101	North Sea	III	-	-	-	97.6	-	-	AY546581
UK-H17/5/93	North Sea	III	-	-	-	99.2	-	-	AY546630

JF00Ehi1	Japan	IV- a	92.3	93.7	93.0	92.7	70.5	96.4	AB490792
MI03GL	USA	IV- b	92.1	-	-	94.1	-	-	DQ427105/DQ401193

^a^The highest percent amino acid identity observed for each protein is highlighted in bold

^b^Accession numbers are listed for complete genomes when available, and for the individual nucleotide sequences when not

^c^Gaps in the alignment

Variation in aa sequence of the G protein is found throughout the length of the protein, but with little variation in the putative transmembrane regions. The TMpred program http://www.ch.embnet.org/software/TMPRED_form.html was used for prediction of transmembrane regions and orientation in the G protein of FA28.11.09. The most strongly supported model suggested transmembrane regions between aa 1 - 18 and aa 462 - 483 where the latter has the highest support.

The last ORF (1984 aa) in the VHS virus genome is the large (L) protein that encodes the RNA-dependent RNA polymerase. The L protein from FA28.11.07 is highly similar to the other L protein genes sequenced from other VHS virus isolates, with the exception of one isolate of genotype I, isolate FR-07-71 (Accession nos: AJ233396 and AJ009814) from France. The conserved domains III and IV containing the four major motifs A, B, C and D localized between aa 566 and 790 [cf [21,20]] are also present in isolate FS28.11.09. A putative ATP biding site, with sequence GEGVRG - (20 aa) - K in position aa 1223 to aa 1249, is present in FA28.11.07 and the genotype I isolates with the exception of isolate FR-07-71 from France. The ATP binding site in FR-07-71 and isolates in genotype IV are GEGVRR - (20 aa) - K and GEGIRG - (20 aa) - K, respectively.

Ten amino acid residues that may play a role in the determination of virulence in genotype I isolates have been identified [19] (table 3). Compared to the genotype I isolates, the VHS virus isolate FA28.11.09 genotype III from rainbow trout in Norway, share 2 and 6 amino acids with the avirulent and virulent strains, respectively. No information is available about the N, P and L proteins from other genotype III isolates. It has also been suggested that two regions, related to fusion activity, within the G protein may play a role in determination of virulence [22]. The VHS virus isolate, FA28.11.09, from rainbow trout in Norway shares six out of seven amino acids, believed to be important for determination of virulence, with the highly virulent FR-07-71 strain (table 4). However, so do all other VHS virus genotype III isolates (see accession numbers in table 2).

**Table 3:** Amino acid residues that may play a role in the determination of virulence [19] when challenging rainbow trout.

	N				P			G	L	
Position	82	83	371	392	39	41	78	506	1012	1465
Amino acid	G-E	M-T	R-L	E-G	P-T	E-G	L-F	M-T	I-F	I-L
FA281107	E	A	K	E	T	G	F	M	F	L
Other GIII	-	-	-	-	-	-	-	M/V	-	-

The virulent strains are, Hededam and FR-14-58, are isolated from rainbow trout, while the avirulent strains, UK-96-43 and DK-M.rhabdo, are from herring and cod. Position = the position of the amino acid residues within the respective proteins. The first amino acid in each column was conserved among avirulent strains and the latter among virulent strains. The VHS virus isolate (FA28.11.09) from rainbow trout in Norway share 2 and 6 amino acids with the avirulent and virulent strains, respectively. No information is available about the N, P and L proteins sequences from other genotype III isolates.

**Table 4:** Amino acid residues in the G protein that may play a role in the determination of virulence [22].

	G protein residues						
	118	135	139	140	161	431	433
FR-07-71	Q	T	S	K	K	L	I
FR-07-71 mutants			I/N	R	R		T
Tr25	R	I	R	K	K	P	I
Tr25 mutants			N	N/E			
FA281109	Q	A	S	K	K	L	I
Other GIII isolates	Q	A/T	S	K	K	L	I

The FR-07-71 VHS virus isolate is highly virulent, while the mutant (07-71 mutant), Tr25 (an attenuated laboratory variant of FR-07-71), and Tr25 mutants have a low virulence. The VHS virus isolate (FA28.11.09) from rainbow trout in Norway share six out of seven amino acids with the virulent FR-07-71 strain.

### Phylogeny

**Figure 3:**
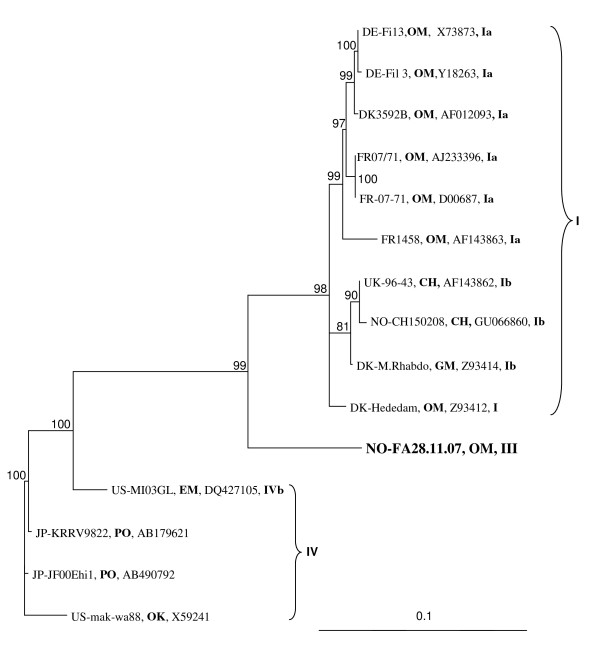
**The phylogenetic relationship of the VHS virus isolate (FA28.11.07) from rainbow trout collected in Norway in 2007 based on the complete sequences of the N protein ORF**. A VHS virus form herring (CH18.03.08) collected in the same area is also included. Phylogram resulting from maximum-likelihood analysis in TREE-PUZZLE (quartet-puzzling). The scale bars shows the number of substitutions as a proportion of branch lengths. **CH **= *Clupea harengus*, **EM **= *Esox masquinongy*, **GM **= *Gadus morhua*, **OK **= *Oncorhynchus kisutch*, **OM **= *O.mykiss*.

**Figure 4:**
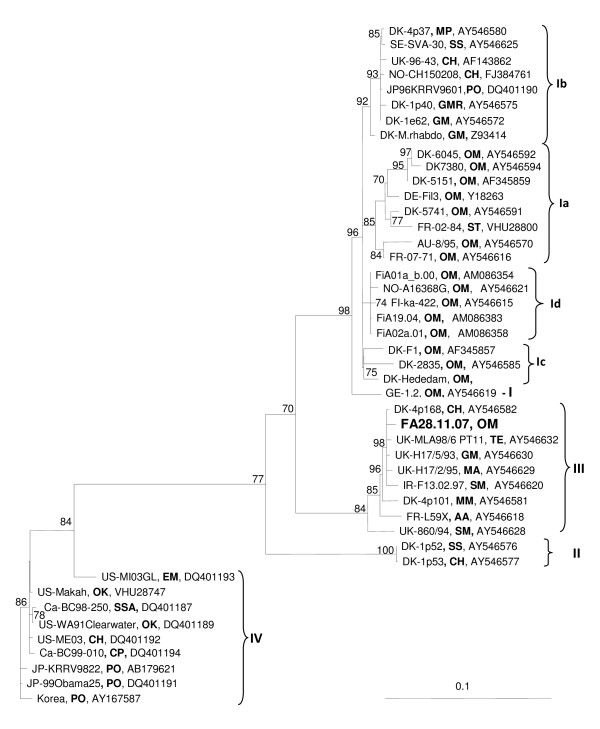
**The phylogenetic relationship of the VHS virus isolate (FA28.11.07) from rainbow trout collected in Norway in 2007 based on the complete sequences of the G protein ORF**. FJ384761 and AY546621 are VHS viruses from Norway. Phylogram resulting from maximum-likelihood analysis in TREE-PUZZLE (quartet-puzzling steps). The scale bars shows the number of substitutions as a proportion of branch lengths. **AA **= *Anguilla anguilla*, **CH **= *Clupea harengus*, **CP **= *Clupea pallasii*, **MP **= *Micromesistius poutassou*, **EM **= *Esox masquinongy*, **GM **= *Gadus morhua*, **GMR **= *Gaidropsaurus mediterraneus*, **MA **= *Melanogrammus aeglefinus*, **MM **= *Merlangius merlangus*, **OK **= *Oncorhynchus kisutch*, **OM **= *O.mykiss*, **SM **= *Scophthalmus maximus*, **PO **= *Paralichthys olivaceus*, **SS **= *Sprattus sprattus*, **SSA **= *Salmo salar*, **ST **= *Salmo trutta*.

Analyses of the relationship of the VHS virus isolate FA28.11.07, based on nucleotides of the complete open reading frame (ORF) of the N (1215 nt) and G (1524 nt) proteins, show that this isolate belong to genotype III (Figures 3 and 4). The closest relatives, based on the ORF of the G protein, are VHS virus isolates from Atlantic cod, Norway pout, and haddock collected in the North sea and herring and turbot collected in Skagerrak and Ireland, respectively. The genotype, GIII, constitutes a sister group to GI in both phylogenies. The nucleotide sequence of the G protein from FA28.11.07 is identical to that published (Accession no:EU547740) by Dale et al [9]. The VHS virus from herring (CH15.02.08), collected in the mouth of Storfjorden, belongs to genogroup Ib. The rainbow trout isolate from Norway (FA28.11.07) is the only fully sequenced member of the GIII. The phylogeny based on the nucleotide sequences of the ORF of the N protein shows stronger support values compared to a similar analysis using the G gene, however, this could be a result of the choice of isolates and the number of isolates included in the two phylogenies.

### Challenge experiment

The rainbow trout used in the challenge experiment, came from fresh water and were put directly in full sea water, where they suffered some mortality (8.8%) during the acclimatization period. Most of the mortality seemed to be due to poor smoltification, but bacteria (*Vibrio *spp. and *Aliivibrio *spp.) were isolated from a few fish (Accession nos: EU862328, EU862329, EU862330, EU862331, EU862332, EU862333, EU862334) and IPN virus was present in all of the fish. The dominating bacteria were *Vibrio splendidus*-like. The mortality stopped one week before the start of the experiment. However, the fish were still positive for IPN virus at the time of challenge, i.e. they were carriers of the virus (Ct values above 30). The fish remained positive for IPN virus throughout the experimental period and a few fish were also positive for *Vibrio *spp and *Aliivibrio *spp. All fish tested before the start of the experiment were negative for VHS virus.

**Figure 5:**
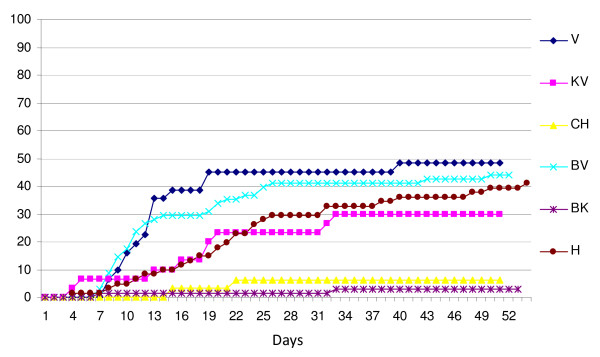
**Percent mortality in the different groups during experimental period**. V = i.p. challenged rainbow trout, *O. mykiss *(isolate FA28.11.07), KV = co-habitants with the i.p. challenged *O. mykiss *(isolate FA28.11.07), BV = bath challenged *O. mykiss *(isolate FA28.11.07), H = group bath challenged with homogenate from VHS virus positive *O. mykiss*, BK = control group for the two groups of bath challenged *O. mykiss*, and CH = rainbow trout i.p. challenged with VHS virus from herring (CH18.03.08).

The mortalities in the different groups varied from 2.9% in the bath control group, BK (N = 68), and up 48.4% in the i.p. challenged group, V (N = 31) (Figure 5). The mortality in the bath challenged group (BV, N = 68) was 44.1% while the mortality among the co-habitants (KV, N = 30) was 30%. The total mortality in the tank challenged by homogenate from rainbow trout (group H, N = 61) was 41%. The group that was challenged with homogenate from VHS virus positive herring (group CH) suffered 6.3% mortality.

**Figure 6:**
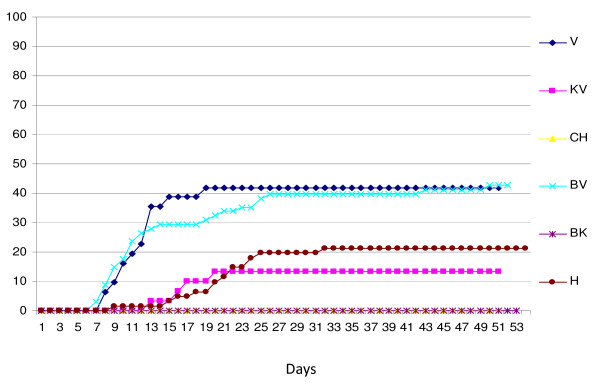
**Percent mortality in the different groups during experimental period excluding rainbow trout that was negative for presence of VHS virus**. V = i.p. challenged rainbow trout (*O. mykiss*), KV = co-habitants with the i.p. challenged *O. mykiss*, BV = bath challenged *O. mykiss*, H = group bath challenged with homogenate from VHS virus positive *O. mykiss*, BK = control group for the two groups of bath challenged *O. mykiss*, and CH = rainbow trout i.p. challenged with VHS virus from herring (CH18.03.08).

Not all fish that died in the different groups were positive for presence of VHS virus. If the fish that were negative for presence of VHS virus are removed from the mortalities the pattern of mortality remains, however, more or less the same (Figure 6). The mortalities associated with presence of VHS virus in the V and BV were 41.9% and 44.1%, respectively. None of the fish that died in the control group, BK, or the CH group were positive for VHS virus. We were not able to identify any other pathogens that could explain the mortalities of the fish that were negative for presence of VHS virus.

None of the fish in groups V (N = 11) and KV (N = 16) were positive for VHS virus at the termination of the experiment 53 days post challenge, while 2 and 3 fish out of 33 and 37 fish examined were positive in groups BV and H, respectively. These five fish were carriers of VHS virus (ct values > 35) and did not show any signs of disease. The virus was present in kidney, heart and spleen tissues, while the brain from one fish only was positive. Of the 97 fish sampled at the termination of the experiment 53 days post challenge 5.2% were carriers of the VHS virus.

The amount of VHS virus template in the kidney and brain of fish in the two challenge groups V and BV have been quantified using the elongation factor alpha as a standard. The kidney tissue from 13 individuals in group V was positive for presence of VHS virus template while only 10 individuals had positive brain tissue (CNS). In the BV group 28 and 27 individuals had positive kidney and CNS, respectively. Only one fish was found to be positive for VHS virus 20 days after injection of the virus (group V), while in the bath challenged group nine fish were positive. An individual sampled 12 days after challenge in group V had the highest expression of VHS virus genome/mRNA. This expression was 6.7 million times higher compared to the lowest expression (sampled 9 days post challenge) of these templates in positive kidney tissue. The individual, in the V group, with the highest expression of VHS virus template in the CNS was sampled 7 days post challenge. None of the fish in this group had positive CNS after 18 days post challenge. In the bath challenged group, BV, the highest expression of VHS virus templates in kidney tissue was found 11 days post challenge while the highest expression in the CNS was seen 42 days post challenge. The latter specimen had negative kidney tissue. Of the nine fish that were positive after day 20 post challenge five had positive kidneys and 8 had positive CNS.

VHS viruses were isolated from all challenged groups except the control group and the CH group. Partial sequences of the genome showed that the reisolated viruses were identical to the FA28.11.07 isolate (Accession no: BV group: FJ362510, FJ362511, H group: FJ362512, FJ362513, KV group: FJ362514, V group: FJ362515).

### Pathology

**Figure 7:**
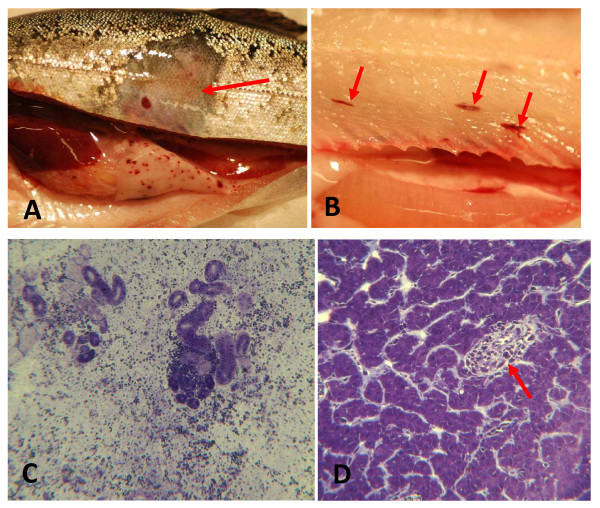
**Pathology**. A) Rainbow trout from the co-habitation group (KV) collected 12 days after challenge. Note the loss of scales and epidermis (arrow) and haemorrhages on the *vicera*. This individual was positive (kidney; Ct = 26) for presence of VHS virus. B) Rainbow trout from the bath challenged group (BV) collected 7 days after challenge showing haemorrhages (arrows) in the somatic muscle (kidney, VHSV Ct = 27). C) Loss of haematopoietic cells and massive haemorrhages in the kidney of a rainbow trout (KV group) collected 18 days after challenge (kidney; VHSV Ct = 36, spleen; VHSV Ct = 28), D) Accumulation of inflammatory cells (arrow) surrounding a blood sinus in the liver of rainbow trout (group BV) collected 19 days after challenge (kidney, VHSV Ct = 24).

The weight and length of the fish in the different groups at the termination of the challenge is given in table 5. In all groups, including the control group (BK), some rainbow trout showed loss of scales and skin ulcers. In the two groups that were bath challenged (groups BV and H) a few fish had haemorrhages on the viscera (figure 7A). A few fish in all VHS virus challenged groups, V, KV, H and BV, showed corkscrewing and had eye and somatic muscle bleedings (figure 7B), pale gills, slight epicarditis and some necrosis of heart myofibers in the ventricle. The most pronounced changes were seen in the kidneys which were slightly swollen with marked necrosis, haemorrhages and loss of haematopoietic cells (Figure 7C). Only minor changes were seen in the liver of strongly positive fish (Figure 7D).

**Table 5:** Mean weight, length, and Hct of the experimental fish in the different groups at the termination of the experiment 53 days post challenge.

Group	N	Weight	Length	Hct
**V**	11	115.1	20.7	61.3
**KV**	16	133.2	21.6	66.9
**H**	35	111.9	20.9	54.3
**BV**	33	108.8	20.6	56.8
**BK**	29	112.4	20.9	58.1
**CH**	23	122.7	21.7	62.2

The mean haematocrit values from moribund fish positive for VHS virus in groups V, KV and H were about 23.0 and for those in group BV it was 12.5. In the control group BK and the challenged group CH the mean haematocrit values were about 55.0 in fish sampled before day 25 after challenge, while at the termination of the experiment the values were 58.1 (N = 29) and 62.2 (N = 23), respectively. One moribund fish in group BK, collected 32 days after start of the experiment, had a haematocrit value = eight. The haematocrit values in all groups at the termination of the experiment are presented in table 5.

## Discussion

### Genome

The complete genome of VHS virus isolates belonging to genotypes I [19,20] and IV (accession no: AB490792) have already been published and this study presents the first complete sequence of the coding region of a genotype III isolate (FA28.11.07). Like all other members of the genus Novirhabdovirus the VHS virus genotype III has the same gene arrangement and similar intergenic regions (ITRs) with polyadenylation signals and transcription initiation sites [19,20,23-25]. There is little variation in the length of the ITRs within the VHS virus species, and the conserved motifs (A, B, C and D) in the L protein [cf [20]] are the same for all VHS viruses sequenced. The ATP binding site in FA28.11.07 is consistent with the consensus sequence for ATP binding sites found in a number of protein kinases and in other negative sense RNA virus polymerases [21].

The distribution of VHS virus genotype III in the North Sea and the North Atlantic ocean has been well documented [13,26-28]. Species like Atlantic Herring *(C. herrengus)*,Norway pout *(T. esmarkii) *and predators of these species, like cod *(G. morhua) *and haddock *(M. aeglefinus) *could carry the virus close to aquaculture facilities. Virulence factors for the VHS virus have not been identified, but it has been shown that the genetic difference between virulent freshwater strains and avirulent marine strains can be very small [19]. VHS virus, like all reproducing units (based on RNA or DNA), consists of populations individuals (virions) that vary in genotypes and appear in a mutation-selection balance. This natural variation will increase as a result of mutation and mutation rates may be high in RNA viruses. The mutation rate of VHS virus genotype III in natural populations is, however, not known. The different variants constituting a virus population consist of highly related virions that may have different phenotypical properties [29]. Hence, when a farmed population of rainbow trout is exposed to a population of marine VHS viruses the variant best adapted to this new host will dominate and may cause disease [3]. The virus may also mutate after it has infected rainbow trout, but there can be no replication followed by mutations unless the virus is able to infect and multiply in rainbow trout. Hence, the VHS virus detected in the rainbow trout farm in Storfjord must have been "pre-adapted" to this fish species, while the VHS virus from herring (CH15.02.08), collected in the outlet of Storfjorden, was not able to establish an infection in rainbow trout.

### Challenge experiment

The fish used in the challenge study suffered a low mortality the first two weeks after arrival, but the mortality ceased one week prior to the challenge. A single cause of these mortalities was not identified, but several factors could have played a role. The fish were taken from a fresh water site, and put directly in full seawater and this stressful event was probably the main cause for the mortalities. However, among the mortalities were fish positive for bacteria and IPN virus. These agents may have affected the mortality observed in the period before start of the challenge and during the experimental period. The IPN virus detected before challenge was also present in the fish throughout the experimental period, but at a low level (carrier state). IPN viruses are very common in the production of salmonids in Norway and it is not known if these virus infections may interfere with infections with VHS virus.

It has been shown in virulence studies of VHS viruses that the challenge method is important for the resulting mortality [12]. Using marine isolates of VHS virus they found that immersion did not cause any mortality while some of the same isolates caused mortality when injected. The mechanism behind this has been studied by Brudeseth et al [30], who found inefficiency at infecting rainbow trout to correlate with a weak ability of the virus to translocate over polarized, primary GEC cultures and a low level of *in vitro *infectivity of VHS virus isolates in primary cell cultures. The present study shows that rainbow trout in full sea water suffers a moderate mortality (about 40%) after exposure to the marine VHS virus genotype III isolate, FA28.11. 07, irrespective of challenge method (immersion or injection). However, the mortalities obtained in this study are relatively high (regardless of infection route), compared to previous studies where rainbow trout has been challenged with genotype III isolates of VHS virus [12,31], but much lower than observed by Dale et al [9]. In the latter study [9] 10.1 gram rainbow trout fingerlings were challenged, using a VHS virus isolate from the same farm as the isolate used in this study, and the resulting mortalities were 100% and 70% after intraperitoneal injection and immersion, respectively. This is very different from the moderate mortality seen in this study where the mortality in the bath challenged group (BV) was slightly higher than in the ip challenged group (V). In the present study the conditions were full seawater and fish with a mean weight of 47.4 grams (at the start of the experiment), hence, the conditions were as close as possible to that in the marine farm where the outbreak occurred and the virus was isolated. In our opinion the use of rainbow trout fingerlings in fresh water [9] is not a suitable system for challenge experiments using marine VHS virus isolates with the aim to obtain knowledge about susceptibility and virulence in marine farms. The use of fingerlings was originally implemented for the study of VHS virus genotype Ia which normally affects rainbow trout fingerlings in freshwater production in Europe. The genotype Ib isolate (CH15.02.08) from herring did not result in any mortality and the virus did not replicate in the rainbow trout.

The fish in the V and BV group in this experiment, were challenged with a high dose (i.p. injection TCID_50 _= 0.5 × 10^8 ^TCID_50_/fish and bath TCID_50 _= 0.8 × 10^8 ^TCID_50_/ml), which is exceedingly higher than what has been reported by other studies infecting rainbow trout with marine genotypes [9,12,31,32] and challenge experiments of other species [13-16,33-35]. This high dose may have contributed to the mortality seen in this study, however, the fish in the H group had approximately the same cumulative mortality as the BV group, and this fish was bath challenged in tissue homogenate with a low amount of VHS virus. Hence, the VHS virus isolate, FA28.11.07, seems to be more virulent for rainbow trout compared to other VHS viruses isolated from marine fish including the isolate (CH15.02.08) from herring collected in the same area. It remains to be shown if this isolate (FA28.11.07) is present in wild fish in western Norway or if the virus has adapted to farmed rainbow trout with a resulting increased virulence.

It has been reported that VHS survivors could become lifetime carriers of the virus, and that they may function as reservoirs for further transmission of the virus [36]. In this study the number of VHS virus positive fish was few at the termination of the experiment. At day 53 only 5 out of a total of 96 survivors from all groups, were positive for VHS virus. These five fishes all had high Ct-values suggesting that a low amount of VHS virus was present in the tissues. These fish that could possibly become carriers, all belonged to the two groups that were bath challenged. The groups challenged by i.p. injection with cultured virus and their cohabitants seemed to rid themselves of the virus. The fate of the possible carrier fish was not determined. However, there could be three possible outcomes; I) the fish will eventually develop disease and die, II) they may continue as carriers or III) they may rid themselves of the virus. The statement that VHS virus infections may lead to lifelong latent infections in surviving fish, which later may infect new hosts [36], must be considered in relation to other studies that report limited viral recovery in surviving fish after challenge experiments with VHS virus [15,37]. Real time RT-PCR analysis of 60 rainbow trout from the affected farm in Storfjorden, Norway, three months after the outbreak of disease, failed to detect VHS virus in kidney and CNS of the fish sampled (Vidar Aspehaug, pers com). Hence, it is a possibility that surviving rainbow trout, after a challenge with the VHS virus genotype III isolate FA28.11.07, are able rid themselves of this virus. To confirm this it will be necessary to follow the fish over a longer period.

### Virulence

According to Gaudin et al [22] a single mutation at position 139 (S - I/N) in the G protein was enough to lower the virulence of mutant isolates of genotype I, and this was further decreased by an additional mutation at positions 140 (K - R) and 433 (I -T). A third mutation at position 161 (K-R) resulted in the most attenuated phenotype. Mutations at positions 118 (Q - R), 135 (T - I) and 431 (L -P) also seem to lower the virulence of type I VHS virus isolates, and Gaudin et al [22] concluded that simultaneous mutations in two distant regions of the glycoprotein (region I aa 135 - 161 and region II aa 431 - 433) could give maximal attenuation of virulence. The FA28.11.07 isolate, presented in this study, which has an identical nucleotide sequence with another isolate from this farm [9], shares all but one amino acid (position 135) with the highly virulent FR-07-71 isolate. At this position the FA28.11.07 isolate has an alanine instead of the threonine found in isolate FR-07-71. However, FA28.11.07 shares the same amino acids in these positions with other marine genotype III isolates shown to be of low virulence [12,13]. Hence, there is no support for claiming that substitutions at these positions affect the virulence of genotype III isolates.

The genotype III isolate UK-860/94, from farmed turbot in Scotland, showed increased virulence for rainbow trout after five *in vivo *passages in rainbow trout [38]. They were not able to detect any mutations in the sequence of the G-protein gene, but suggested that other genes might have been responsible for the observed increase in virulence [38]. If they are correct there is a possibility that a marine genotype III virus could have been recently transmitted from a marine carrier fish to rainbow trout in aquaculture and, in a short time, evolved increased virulence. It has been suggested that 10 amino acid positions in four proteins (N, P, G and L), may be virulence markers [19]. The FA28.11.07 isolate shares the majority (six) of the amino acids with the virulent fresh water strains of genotype I isolates and only two amino acids with the avirulent marine strains. Hence, it can be concluded that the genotype III VHS virus isolate FA28.11.07 shares many of the amino acid residues with that of highly virulent fresh water genotype I isolates. However, no information is available about these amino acid positions in the low virulent strains within genotype III. Considering the relatively high mortality obtained after bath challenge in the present study, compared to similar experiments with other marine genotype III VHS viruses, the factors influencing virulence have yet to be identified.

## Conclusions

Based on analyses of the nucleotide sequences of the complete ORF of the N and G proteins it can be concluded that the VHS virus isolate FA28.11.07 is a new, distinct, isolate belonging to genotype III, and being moderately virulent to rainbow trout [cf [3-5,11,32,39]]. Only future research can show if this VHS virus isolate from rainbow trout in Storfjorden also exits in natural populations of marine fish in the fjord or if the isolate represents a new adaptation to rainbow trout.

## Materials and methods

In November 2007 a viral haemorrhagic septicaemia virus (VHSV), genotype III (Accession nos: EU336985, EU481506), was isolated from rainbow trout (*Oncorhychus mykiss*) suffering mortality in a marine farm in Storfjorden at the northern west coast of Norway [10]. The VHS virus isolate has been named FA28.11.07, and the third passage of this isolate was used to challenge rainbow trout in full sea water. Nearly identical VHS virus isolates were found in two neighboring rainbow trout farms (isolate FA28.02.08S, accession nos: GU121099, GU121100 and isolate FA28.02.08V, Accession nos: GU121101, GU121102), and in July 2008 this virus was also found in a more distant farm in the same fjord. All farms are owned by the same company.

In addition to VHS virus type III, the rainbow trout in the first farm were infected by *Candidatus *Piscichlamydia salmonis, an unidentified chlamydia-like species, *Flavobacterium psychrophilum*, and a new species of microsporidia, *Paranucleospora theridion*. It is not known to what extent these pathogens may have contributed to the mortality or to what extent the mortality was caused by the VHS virus. However, after screening with real time RT PCR (primers and probe described below), only 2 out of 30 moribund rainbow trout were found to be positive for presence of VHS virus on the collection date 28 November 2007. The number of VHS virus positive fish increased to 50% in the middle of December 2007, while it was not possible to detect the virus in kidney and brain tissues from fish (N = 60) sampled in March 2008 (V. Aspehaug, pers. com.)

A VHS virus isolate was also obtained from herring (*Clupea harengus*) collected at the outlet of Storfjorden, but this isolate (CH18.03.08) belonged to genogroup Ib (Accession nos: FJ384761, GU066860).

### Challenge experiment

Rainbow trout, with a mean weight and length of 47.4 gram and 15.7 cm, were taken into the research facility, Industrilaboratoriet, at the University of Bergen in January 2008. The fish came from a fresh water site in western Norway and were put directly in full sea water (34‰). A low mortality was registered during the first two weeks after arrival and some of the fish had skin ulcers. Bacteria, *Vibrio *spp were isolated from a few of the moribund fish during the first two weeks after arrival. However, the major loss of fish was probably due to poor smoltification, and the mortality stopped a week before the fish were used in the challenge experiment. A subsample of fish (N = 40) were checked for presence of VHS virus and IPN virus, using real time RT PCR assays (see below), before the start of the experiment. None of the fish that died during the acclimatization period were positive for VHS virus, but they were all carriers of the IPN virus and a few were positive for different bacteria (*Vibrio *spp. and *Moritella viscosa*). The fish were kept in 5 tanks (0.15 m^3^) with running sea water (34‰) at mean temperature of 10°C.

The challenge experiment was designed to see if the VHS virus isolate, FA28.11.07, may cause mortality and if different challenge methods may influence mortality. The wild type VHS virus from herring (CH15.02.08) was used as a control.

#### Tank 1

This tank was used to test the effect of intraperitoneal injection (ip) of the VHS virus isolate FA28.11.07 and the effect of transmission from infected to non-infected rainbow trout (co-habitation effect). Rainbow trout, N = 31 (code = V), were injected intraperitoneal (ip) with 0.2 ml of supernatant from cell culture with TCID50 of 1.0 × 10^9^/ml, ie. giving a final challenge dose of TCID50 = 1.1 × 10^6^/gram fish tissue (or TCID50 = 0.5 × 10^8^/fish). In addition to the ip challenged fish 30 rainbow trout (code = KV) were added as co-habitants. A total of 61 rainbow trout were kept in tank 1.

#### Tank 2

This tank contained 68 rainbow trout (code = BV) that were challenged by a bath consisting of 15 ml of the VHS virus isolate FA28.11.07 (TCID50 = 1.0 × 10^9 ^VHS virions/ml) in 20 liter sea water for 30 minutes, i.e. at a concentration of TCID50 = 0.8 × 10^6^/ml.

#### Tank 3

This tank contained 68 rainbow trout (code = BK) that were bathed in 15 ml cell culture media in 20 liter sea water for 30 minutes. This group constituted the control group for the bath challenged rainbow trout.

#### Tank 4

This tank was used to test if VHS virus from rainbow trout carriers could be transmitted by bath challenge. Rainbow trout, N = 61 (code = H), were bathed for one hour in 20 liters of sea water added 45 ml of sterile filtered homogenate (0.2 μm) of gill and kidney tissues from rainbow trout that were asymptomatic carriers of VHS virus. It was not possible to culture the virus from this homogenate. The virus was only detected by real time RT PCR. The homogenate was obtained from carrier rainbow trout that came from the same marine farm as the VHS virus isolate FA28.11.07.

#### Tank 5

This tank was used to test the possible effect on rainbow trout of a wild type VHS virus detected in herring (CH18.03.08) from the outlet of Storfjorden. Homogenate made from brain and kidney tissue of the herring was intraperitoneal injected into rainbow trout, N = 30 (code = CH). Each fish was injected with 0.2 ml of sterile filtered homogenate (0.2 μm).

### Sampling

Dead and moribund fish were removed from the tanks two times a day, and weight and length were registered. All fish were examined for external and internal signs of disease. To rule out bacterial infections as a cause of mortality, bacterial samples were taken from a selection of dead/moribund fish and fish sampled randomly. Bacteria were isolated from kidney and grown on blood agar plates containing 1.5% NaCl and incubated at 15°C for at least two weeks. When growth was observed on the agar plates the bacteria were identified by sequencing of the 16S gene using a set of general prokaryotic primers targeting this gene (see below).

The following tissues were sampled from all dead/moribund fish; gills, heart, kidney, spleen, and CNS. The tissues were stored at -20°C before being used for reisolation of the VHS virus or RNA extraction. All dead/moribund fish were tested for presence of VHS virus and IPN virus by real-time RT-PCR. To obtain information about tissue tropism the head-kidney, heart, spleen and CNS tissues were tested from 25 fish with respect to presence of VHS virus. Based on the results from the tissue tropism study the kidney and CNS from all fish in the different groups were tested for VHS virus, while only the kidneys were tested for presence of IPN virus. A blood sample, for measuring the haematocrit, was taken from all fish.

Several tissues (skin, gill, heart, kidney, spleen, gut and CNS) were sampled from a few individuals in each group and fixed in Karnovsky for examination of histopathology.

### Culturing VHS virus

RTgill-W1 cells [40] were cultured in 15 cm^2 ^tissue culture flasks (Nunc) at 20°C in Eagles Minimum Essential Medium (EMEM) (Sigma) supplemented with 10% Foetal Bovine Serum (FBS) (10% v/v), L-glutamine (4 mM) and gentamicin (50 μg/ml). The cells were then subcultured for 7-10 days until the tissue flasks were covered with 60-80% confluent monolayer.

Supernatant containing VHS virus isolate FA28.11.08 (second passage) was diluted 1:100 in PBS and incubated for 1 hour at 15°C in cell culture flasks with the monolayer of RTgill-W1 cells. The inoculum was then removed and replaced by supplemented EMEM as described above, but with 1% FBS. The cells were incubated for five days until cytopathic effect (CPE) could be observed. RNA was extracted from infected cells and reverse transcribed into cDNA as previously described [41] and a real time RT PCR assay was used to test for presence of VHS virus.

### Virus titration

RT-gill W-1 cells were seeded in a 96-well tray and allowed to form a monolayer. A 10-fold dilution series of the VHS virus isolate was made in 2% infection medium and added to the monolayers. To each well, 200 μl of each dilution, ranging from 10^1 ^to 10^17^, was added. Each dilution was used in four parallel wells. The 96-well tray was incubated for 10 days, and TCID_50 _(The dilution in which 50% of the cells are dead) was determined by comparing with uninfected cells.

### Re-isolation of virus

RTgill-W1 cells were also used for reisolation of VHS virus from the different fish groups challenged. Tissue homogenates were made from kidney or CNS tissues from selected fish (Codes: V, KV, BV, BK, H and CH) in the different groups and sterile filtered (0.2 μm) before inoculation on the cells. The cells were kept for three passages or until a CPE could be observed. Presence of VHS virus in cell cultures was confirmed by real time RT PCR and sequencing of the PCR products.

### Histopathology

Tissues (skin, gills, heart, head-kidney, kidney, spleen, and brain) collected were fixed by immersion, at 6°C, in a modified Karnovsky fixative where the distilled water was replaced by a Ringers solution. The fixative contained 4% sucrose. Before embedding in Historesin the tissues were dehydrated through graded series of ethanol as recommended by the manufacturer. Semi sections, 1.5 μm thick, were cut on a Reichert-Jung 2050 microtome and stained in toluidine blue.3 Before embedding in EMBED-812 (Electron Microscopy Sciences) the tissues were stained/post-fixed in 1% OsO_4_. Ultrathin sections were cut on Reichert-Jung Ultracut E. The ultrathin sections (30 - 40 nm) were stained for 1.5 hours in 2% aqueous uranyl acetate solution and then stained with lead citrate.

### DNA/RNA extraction

The extractions of RNA from tissues were performed as described by Devold et al. [41]. RNA-pellets were eluted in 100 μl DEPC-water and stored at -20°C until examination by real time RT-PCR.

cDNA synthesis was performed as follows; 5 μl DEPC-water, 1 μl pd(N)_6 _(random hexamers) and 4 μl RNA template, making a total of 10 μl, was incubated at 70°C for 5 min. A RT-mix was made from 5 μl 5 × RT-buffer 5, 1,25 μl DTT (200 mM DL-dithiothreitol), 2,5 μl 10 mM dNTP, 0,5 μl RNasin, 0,15 μl MMLV and 5,6 μl DEPC-water, making a total volume of 15 μl. This RT-mix was added to the 10 μl template/pd(N)_6 _mix making a total volume of 25 μl. This solution was incubated at 37°C for 60 min [41] after which the cDNA was stored at -20°C.

DNA was extracted from all isolated bacteria using the DNeasy DNA Tissue kit (Qiagen) following the manufactures description of protocol. Elution was performed twice in 50 μl 10 mM Tris-HCl, pH = 8.5 to increase the overall DNA yield, and the DNA was stored at -20°C.

### PCR and sequencing

The partial G gene (654 nucleotides) from VHS virus present in tissues of challenged rainbow trout and virus isolated from challenged fish in cell culture, was obtained by PCR using the cDNA synthesized as described above (accession nos: FJ362510, FJ362511, FJ362512, FJ362513, FJ362514, FJ362515). VG1 (5'-ATG GAA TGG AAC ACT TTT TTC-3') and VD3 (5'-TGT GAT CAT GGG TCC TGG TG-3') were used as PCR primers [42]. The nearly complete genome of the VHS virus isolate FA28.11.07 was obtained by using primers targeting the genome of other VHS virus belonging to genotypes I and IV (The primers can be obtained from the authors).

The partial 16S genes from isolated bacteria were obtained by the following primers; EUGB27F and EUGA1518R [43].

PCR products were run on electrophoresis gels for visualization and the products were purified using QIAquick PCR purification kit (Qiagen) as described by the manufacturer. Sequencing was then performed in both directions using ABI PRISM BigDye terminator chemistry (version 2) according to Applied biosystems (ABI). The PCR primers were used for sequencing.

### Real time RT-PCR

In this experiment six real time RT-PCR assays were used (table 6). Primers and a probe able to detect both VHS virus genotype I and III were obtained by a modification of an assay developed by Mike Snow [44]. The assay targets a region of the nucleoprotein of VHS virus, ie. position 194 - 302 in the ORF of the N gene (Accession no: EU481506).

**Table 6:** Real time PCR assays for VHS virus, *Paranucleospora theridion*, *Flavobacterium psychrophilum*, *Candidatus *Piscichlamydia salmonis and a new species of chlamydia from gills of Atlantic salmon.

Target	Code:	Primers/probe	Amplicon size	Reference
VHS virus	VHSV F08	TGT CCG T**K**C TTC TCT CCT ATG TAC T		Modified
	VHSV probe	CTC ACA GAC ATG GG	109 nt	[44]
	VHSV R08	GCC CTG **R**CT G**M**C TGT GTC A		Modified

*Paranucleospora*	PT-F	CGG ACA GGG AGC ATG GTA TAG		
*theridion*	PT-probe	TTG GCG AAG AAT GAA A	59 nt	[49]
	PT-R	GGT CCA GGT TGG GTC TTG AG		

*Flavobacterium*	Flavo-R	TGT AAA CTG CTT TTG CAC AGG AA		
*psychrophilum*	Flavo-probe	AAA CAC TCG GTC GTG ACC	72 nt	Present study
	Flavo-F	GAT CCT TAT TCT CAC AGT ACC GTC AA		

*Candidatus*	Pch-F	TCA CCC CCA GGC TGC TT		
Piscichlamydia	Pch-probe	CAA AAC TGC TAG ACT AGA GT	60 nt	Present study
salmonis	Pch-R	GAA TTC CAT TTC CCC CTC TTG		

New species of	Sch-F	GGG TAG CCC GAT ATC TTCA AAG T		
gill chlamydia	Sch-probe	TCC TTC GGG ACC TTA C	66 nt	Present study
	Sch-R	CCC ATG AGC CGC TCT CTC T		

Elongation factor	EL1A-elaf	CCC CTC CAG GAC GTT TAC AAA		
1 alpha	EL1A-elam1	ATC GGT GGT ATT GGA AC	57 nt	[45]
*S. salar*	EL1A-elar	CAC ACG GCC CAC AGG TAC A		

The internal control assay El-1A targets the Atlantic salmon cellular elongation factor [45]. The assays for detection of *Paranucleospora theridion*, *Flavobacterium psychrophilum*, *Candidatus *Piscichlamydia salmonis and the new chlamydia species, targeting the genes coding for the SSU, are presented in table 6.

Verso™ 1-step QRT-PCR ROX Kit and Absolute™ QPCR ROX Mix were used for the real time RT-PCR assays. Analysis was performed in a ABI 7500 sequence detection system (Applied Biosystems). The reaction was 15 min at 50°C (Reverse Transcriptase step), 15 min at 95°C (Polymerase activation step) followed by 45 cycles of 95°C for 15 seconds (DNA-dissociation) and 60°C for one minute (Annealing and elongation).

### Efficiency and sensitivity

Prior to real-time RT PCR analysis the VHS virus assay was optimized with regards to concentrations of primers and probe. An efficiency test was preformed to test the efficiency of the VHS virus and elongation factor assays in order to be able to perform relative quantification of the amount of VHS virus in the samples. This test was performed by using a tenfold dilution series. The dilution series was made in the concentration ranging from 477.6 ng/μl to 4.776^-06 ^ng/μl for VHS virus assay and 243.6 ng/μl to 2.436^-06 ^ng/μl for the elongation factor assay. Template used in the efficiency test was as described above. The dilution series was analyzed in triplicates using one-step real-time RT PCR. The mean Ct value for each triplicate was calculated and a standard curve was made by plotting Ct values against the serial logarithmic dilutions. The amplification efficiency (E) for the VHSV08 and EF1A assays were calculated using the formula: (10^-1/-slope^)-1 and were found to be E = 0,98394 and E = 0,8952, respectively.

To perform the sensitivity test a twofold dilution series was made from 0,4 ng/μl to 0,0625 ng/μl. 10 replicates were made from each dilution and analyzed using one-step real time PCR. The sensitivity limit of the VHSV08 assay was set as the highest dilution where all 10 replicates were positive. The detection limit, derived from this sensitivity test for the VHSV08 assay, was found to be Ct = 37.0. This implies that Ct values higher than this limit may not be reproducible.

### Relative quantification

The expression of the target VHS virus genome template was calculated using the formula for normalized expression (NE): NE = (E_reference_)^Ct^reference/(E_target_)^Ct ^target, where E = amplification efficiency.

### Phylogenetic analysis

The nearly complete genome of the VHS virus isolate, FA28.11.07, from the farmed rainbow trout kept in Storfjorden western Norway, was sequenced (Accession no. EU481506). The N and G protein genes were aligned with homologous gene sequences from a selected number of VHS viruses already available on the EMBL nucleotide database, including a Norwegian isolate collected from rainbow trout in 1968 (Accession no: AY546621). The VHS virus (CH18.03.08) from herring (Accession nos: FJ384761, GU066860), collected in the outlet of the fjord, was also included in the analysis. To perform pairwise comparisons between the different viruses, the multiple sequence alignment editor GeneDoc (Available at: http://www.psc.edu/biomed/genedoc) was used. Polymorphic regions were manually aligned and compared for both genes. Gaps in the alignment were deleted (the alignment can be obtained from the corresponding author).

Phylogenetic analyses of the data sets were performed using PAUP* version 4.0 [46] and TREE-PUZZLE 5.2 (Available at: http://www.tree-puzzle.de). TREE-PUZZLE reconstructs phylogenetic trees from molecular data by maximum likelihood, and computes maximum likelihood distances and branch lengths. A model corresponding to GTR+I was identified by the Akaike information criterion using the Modeltest 3.6 script [47] in PAUP v4.0 [46], to be suitable for the datasets. In this study 10 000 quartet puzzling (QP) steps were carried out. The QP tree search estimates support values for each internal branch. Branches showing QP reliability from 90 - 100% can be considered very strongly supported. Branches with lower reliability (>70%) can in principle be trusted. The only phylogeny presented in this study is the result of analysis using the GTR matrix in TREE-PUZZLE. Phylogenetic trees were drawn using TreeView [48].

## Competing interests

The authors declare that they have no competing interests.

## Authors' contributions

AN conceived the study. AN, HD and SN planned the experimental design. AN, HD and SN carried out the challenge experiment and sampling. SN cultured the virus and did all the sequencing. HD analyzed all samples. KW did the processing for histology and AN and KW did the histological examinations. KFO sampled the field material and helped with the processing and analyzing of this material. AN and HD drafted the manuscript. All authors critically reviewed and approved the final manuscript.
